# Laparoscopic Transabdominal Preperitoneal Technique for Inguinal Hernia Repair in Adults

**DOI:** 10.7759/cureus.8692

**Published:** 2020-06-18

**Authors:** Nguyen Thanh Xuan, Nguyen Huu Son

**Affiliations:** 1 Department of Abdominal Emergency and Pediatric Surgery, Hue Central Hospital, Hue, VNM; 2 Pediatrics, Hue Central Hospital, Hue, VNM

**Keywords:** transabdominal preperitoneal (tapp), inguinal hernia, prolene mesh, outcome, complication

## Abstract

Background

Inguinal hernia repair is one of the most commonly performed operations in general surgery, especially in the digestive field. Since the introduction of laparoscopic repair as well as using a synthetic mesh, the surgical trends have changed in the last decade in treating inguinal hernias. The laparoscopic transabdominal preperitoneal gives a better view of the inguinal anatomy, and the procedure also has a short learning curve. We aim to evaluate the safety and early outcome of the laparoscopic transabdominal preperitoneal technique for inguinal hernia repair using a Prolene^®^ mesh (Ethicon Somerville, NJ, USA).

Methods

A prospective study was carried out among 31 adult patients with 34 inguinal hernia cases. They underwent the laparoscopic transabdominal preperitoneal technique with a Prolene mesh at the Hue Central Hospital from December 2018 through May 2019.

Results

The mean age was 60.4 ± 11.8, and 96.8% of cases were male. Strangulated hernia and incarcerated hernia accounted for 2.9% and 8.8% of cases, respectively. The mean duration of unilateral inguinal hernia repair and bilateral inguinal repair was 57.1 ± 17.3 minutes and 80.3 ± 10.6 minutes, respectively. The mean duration of the postoperative hospital stay was 3.9 ± 1.4 days. One (3.2%) case with contralateral inguinal hernia was detected intraoperatively. An early and three-month postoperative evaluation showed that 93.5% and 96.8% of cases were categorized as “very good”, respectively. At the three-month evaluation, one case was reported with sensation disorder of the inguinal area, and there was no recurrence.

Conclusions

Laparoscopic transabdominal preperitoneal inguinal hernia repair is a safe and feasible technique. It allows surgeons to explore the opposite site and resolve the combined peritoneal diseases.

## Introduction

Inguinal hernia is a common surgical condition in countries around the world as well as in Vietnam. The disease can occur at any age, with an incidence of around 25% in men and 2% in women [1].

Abdominal wall reconstruction to repair the inguinal hernia using autologous tissue is the first and most widespread method that has been used. However, these types of surgeries, which use autologous tissue, have disadvantages related to suture stretching for large hernias, patients with weak abdominal walls, and patients with bilateral hernias. Therefore, an artificial mesh is considered to strengthen the wall of the inguinal canal [2].

Today, laparoscopic surgery for the treatment of inguinal hernias is widely used due to the less-invasive nature and good outcomes [3]. The most widely used methods in the world today are the transabdominal preperitoneal (TAPP) and the totally extraperitoneal (TEP) techniques [4,5]. TAPP surgery gives a better view of the inguinal anatomy, and the procedure also has a shorter incision as well as learning curves. TAPP could intraoperatively detect the asymptomatic contralateral inguinal hernias and treatment of all types of inguinal hernias even in cases of implication [4,6].

In Vietnam, laparoscopic surgery to repair inguinal hernias has been performed in some surgery centers. In Hue Central Hospital, this surgical technique has been studied and applied in recent years with the TEP, but no researches have been conducted on the TAPP method. We aim to evaluate the safety and early outcomes of the laparoscopic TAPP technique for inguinal hernia repair using a Prolene® mesh (Ethicon Somerville, NJ, USA).

## Materials and methods

Patients

We prospectively enrolled 31 patients with 34 cases of inguinal hernia (3 patients with a bilateral inguinal hernia) to undergo laparoscopic TAPP surgery using Prolene mesh at Hue Central Hospital from December 2018, to May 2019. Informed consent was obtained from all patients before the study. The steps of operative interferences were explained to all patients. The Local Ethics Committee approved all operative procedures. Ethical approval for this study was granted by the Ethical Review Committee Board of Hue Central Hospital.

Inclusion criteria were adult patients with age over 18 years, an inguinal hernia diagnosed based on clinical examination and ultrasound (direct hernia, indirect hernia, a mix of direct and indirect hernia, recurrent hernia, incarcerated hernia, strangulated hernia), and the American Society of Anaesthesiologists (ASA) grades I, II, and III.

Exclusion criteria were patients with strangulated hernia with over six hours delayed hospitalization or with peritonitis; patients with serious background diseases such as progressive Basedow disease, severe diabetes with complications, unstable angina, renal failure, or progressive tuberculosis; and patients with increased abdominal pressure due to ascites or peritoneal dialysis.

Surgical technique

**Figure 1:**
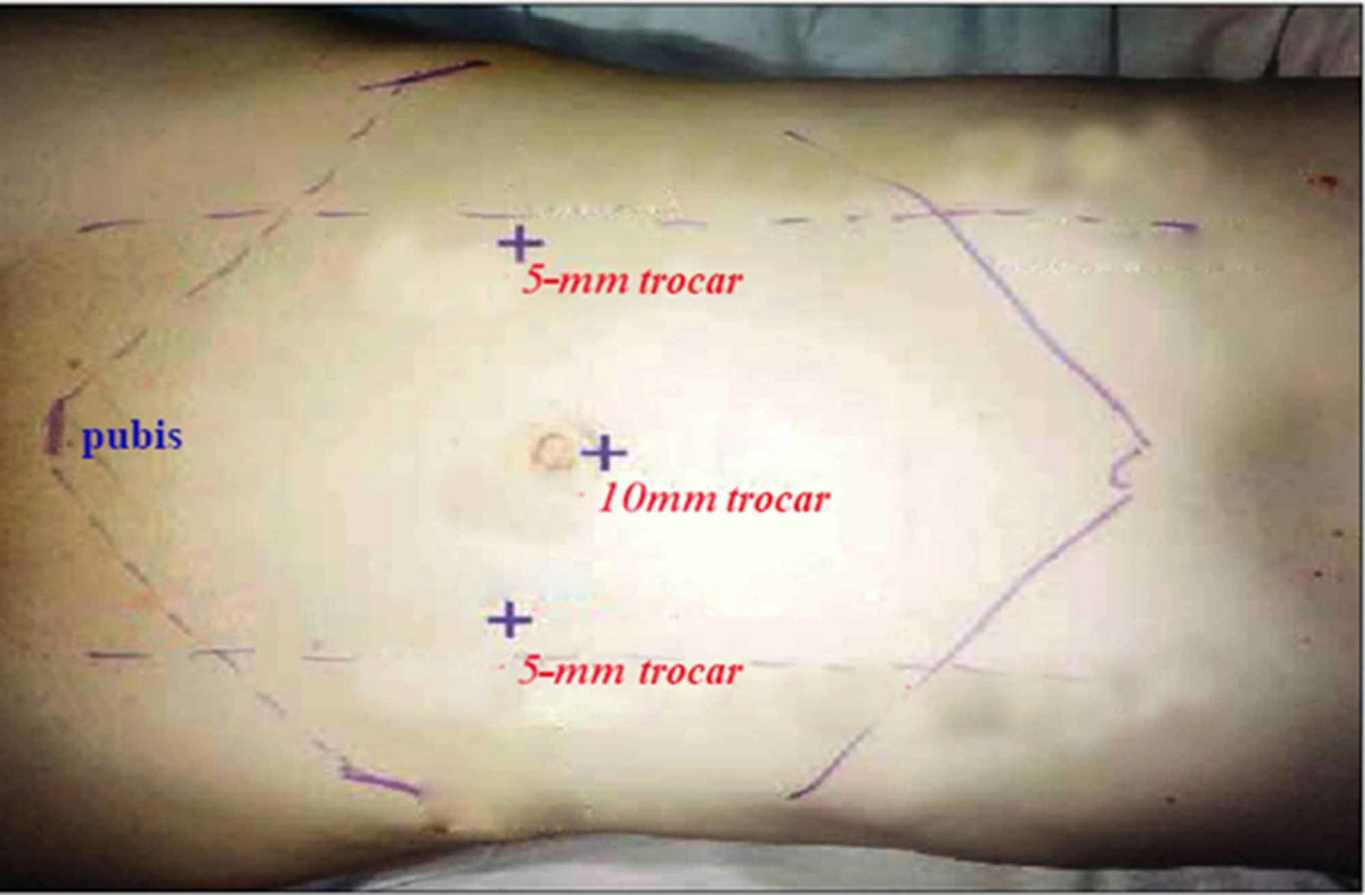
Location of trocar insertion

Under endotracheal anesthesia, the patient was placed on the operating table in a supine position. Initially, we placed a 10-mm trocar into the peritoneal cavity above or below the navel and inserted surgical microscopes into the peritoneal cavity to observe and assess the location of the hernia. We defined the hernia type, measured the size of the deep inguinal hole, and assessed the position of the outside position if any. Two 5-mm trocars were placed on the outer edge of the rectus abdominis in line with the 10-mm trocar position (Figure 1). Next, we proceeded with the dissector through the two 5-mm trocars.

**Figure 2:**
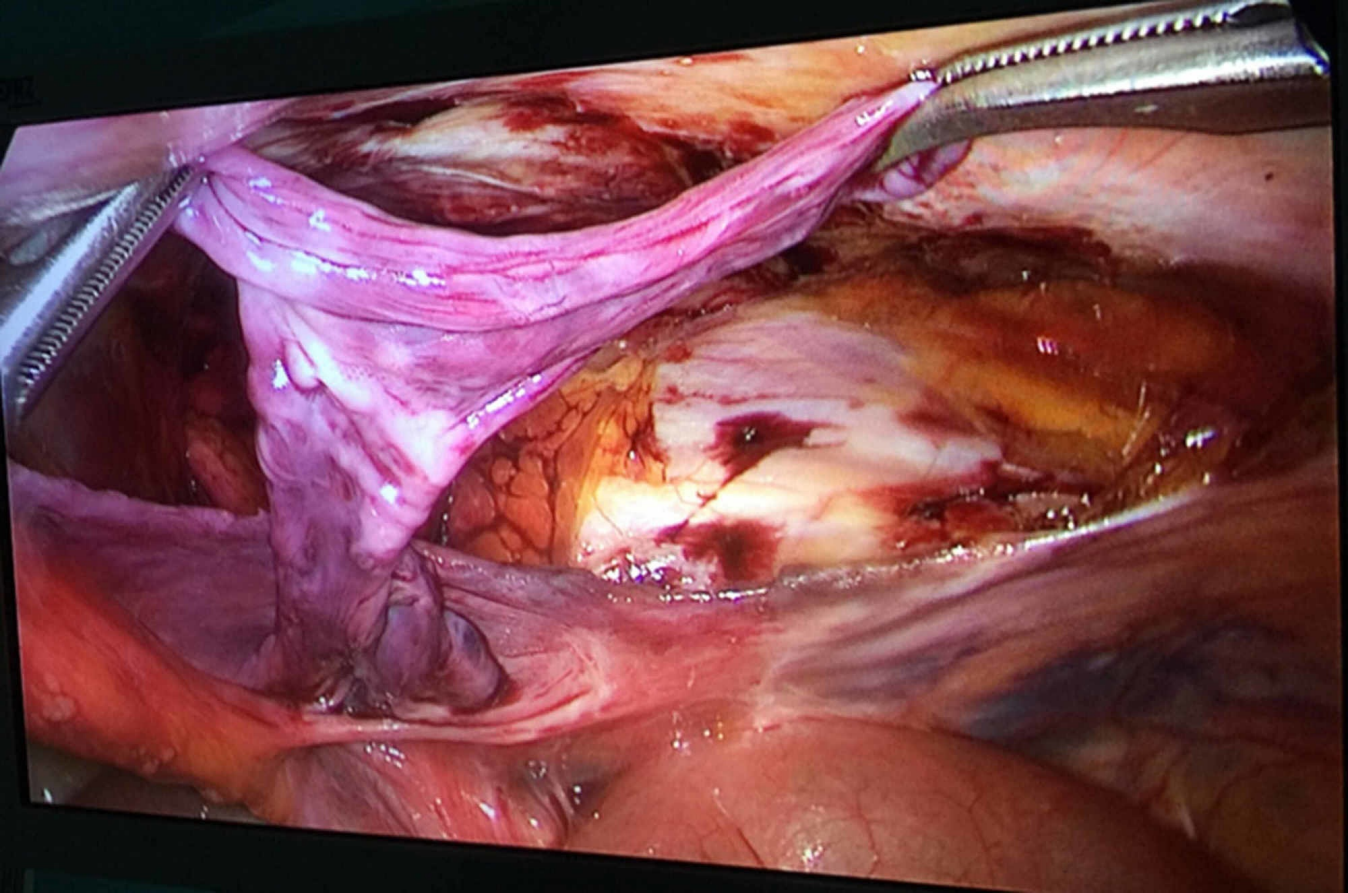
Dissection of the indirect herniated sac

The hernia organ was released before performing on the strangulated or incarcerated hernias. We checked the hernia situation. A peritoneal incision was made from the anterior perianal papillae, about 3-4 cm above the deep inguinal vault dome, from outside to inside to the lateral umbilical fold. Then we separated the peritoneum to the deep inguinal opening, from the bundle of the inferior epigastric vessels, and separated the herniated sac if present (Figure 2).

**Figure 3:**
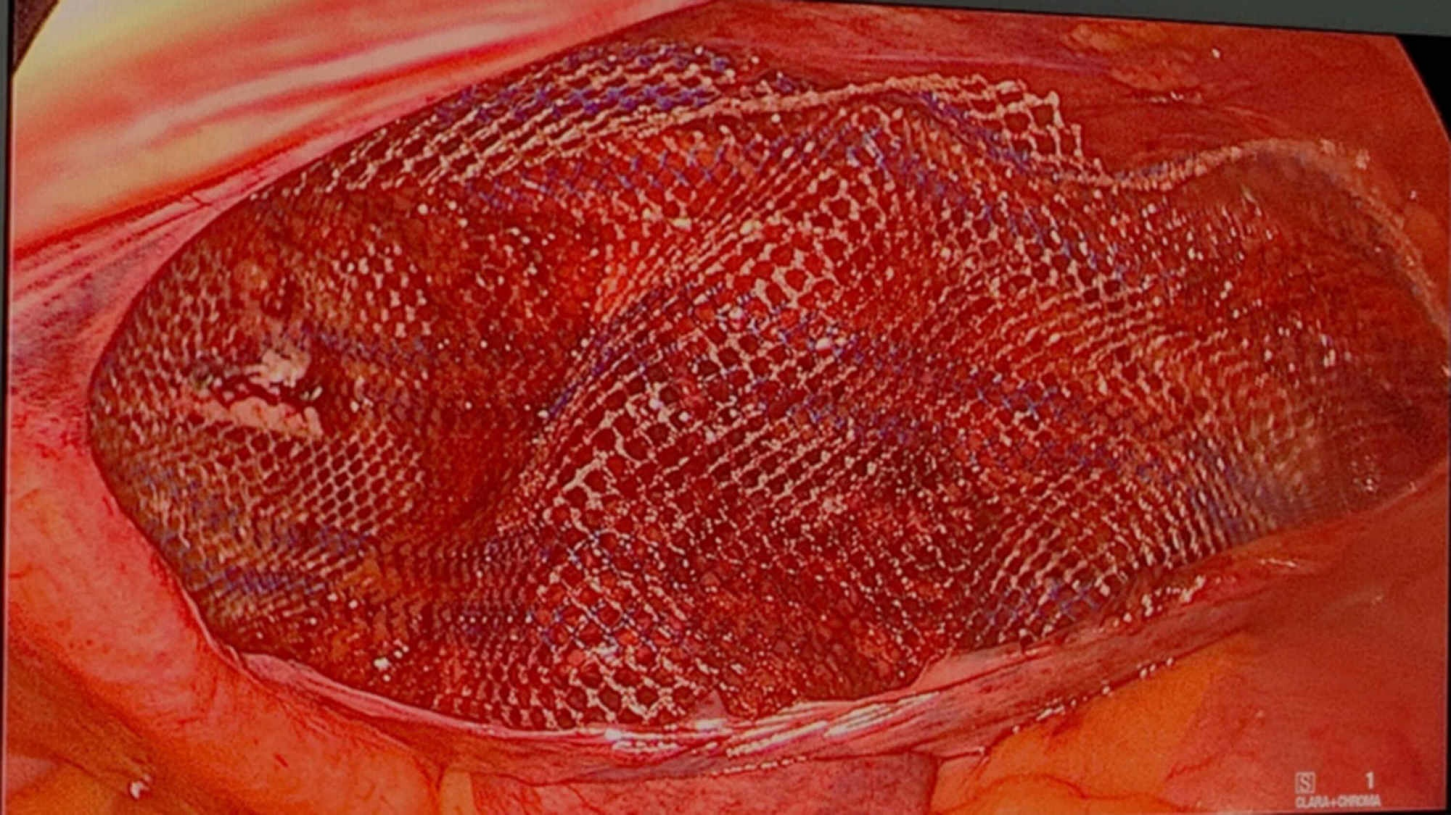
Insertion of the Prolene mesh into the anterior peritoneal cavity

**Figure 4:**
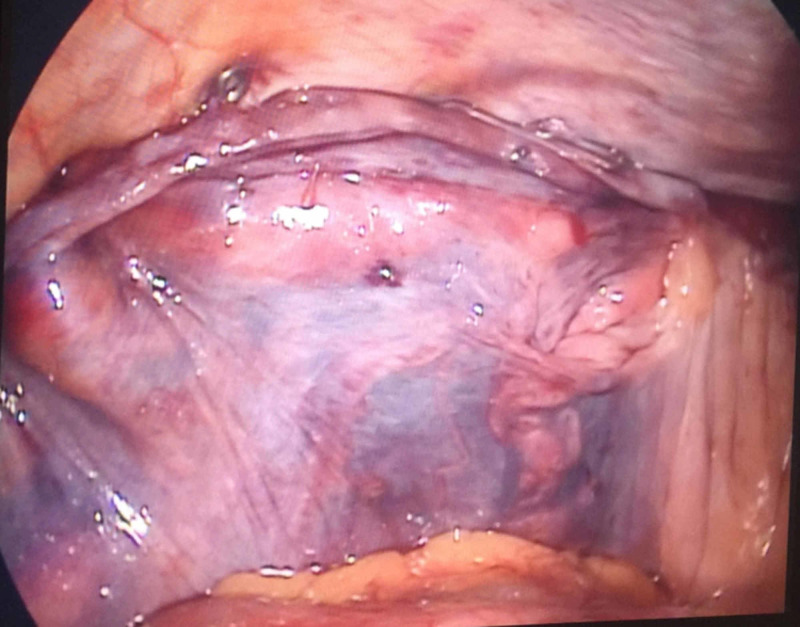
Fixing the artificial sheet with ProTack

A 10 cm x 15 cm Prolene mesh was placed into the anterior peritoneal cavity created (Figure 3). We covered the deep inguinal opening and the posterior inguinal canal. Then we fixed the mesh with ProTack™ (Medtronic, Minneapolis, MN, USA) (Figure 4). Finally, we closed the peritoneum, discharged the gas, and closed the trocar holes.

Evaluation and follow-up

Descriptive data about patient characteristics included age, sex, and clinical and subclinical characteristics of inguinal hernia patients. We classified the inguinal hernias according to the European Hernia Society, by complications. Health classification was defined according to ASA. Postoperative pain was evaluated according to the visual analog scale (VAS) [7]. This scoring system is graded from 0 to 10, with 0 indicating none or no pain, 1-3 indicating mild pain, 4-6 indicating moderate pain, and -10 indicating severe pain.

We evaluated the early outcome after surgery and re-examined after one month and three months. Follow-up data were collected from clinical examinations during a subsequent visit to our outpatient clinic. Daily activity levels were checked to find out when patients resumed their usual work or preoperative daily activities.

Statistical analysis

The data were analyzed using the SPSS Statistical Analysis Program for Windows, Version 20 (IBM Corp., Armonk, NY, USA). We calculated the frequencies of the categorical variables and the means of the continuous variables.

## Results

**Figure 5:**
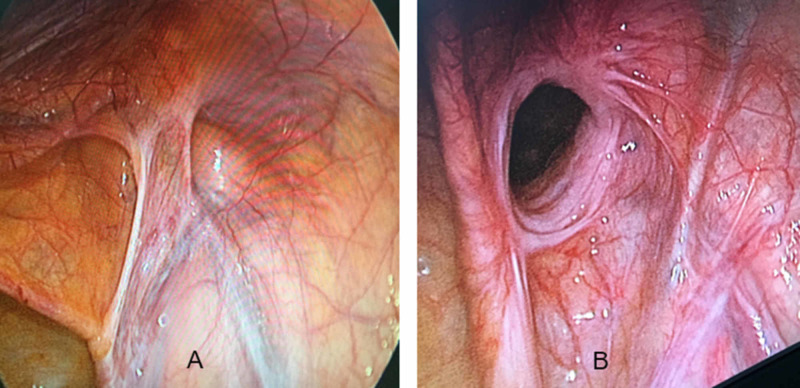
(A) Indirect hernia and (B) direct hernia

**Figure 6:**
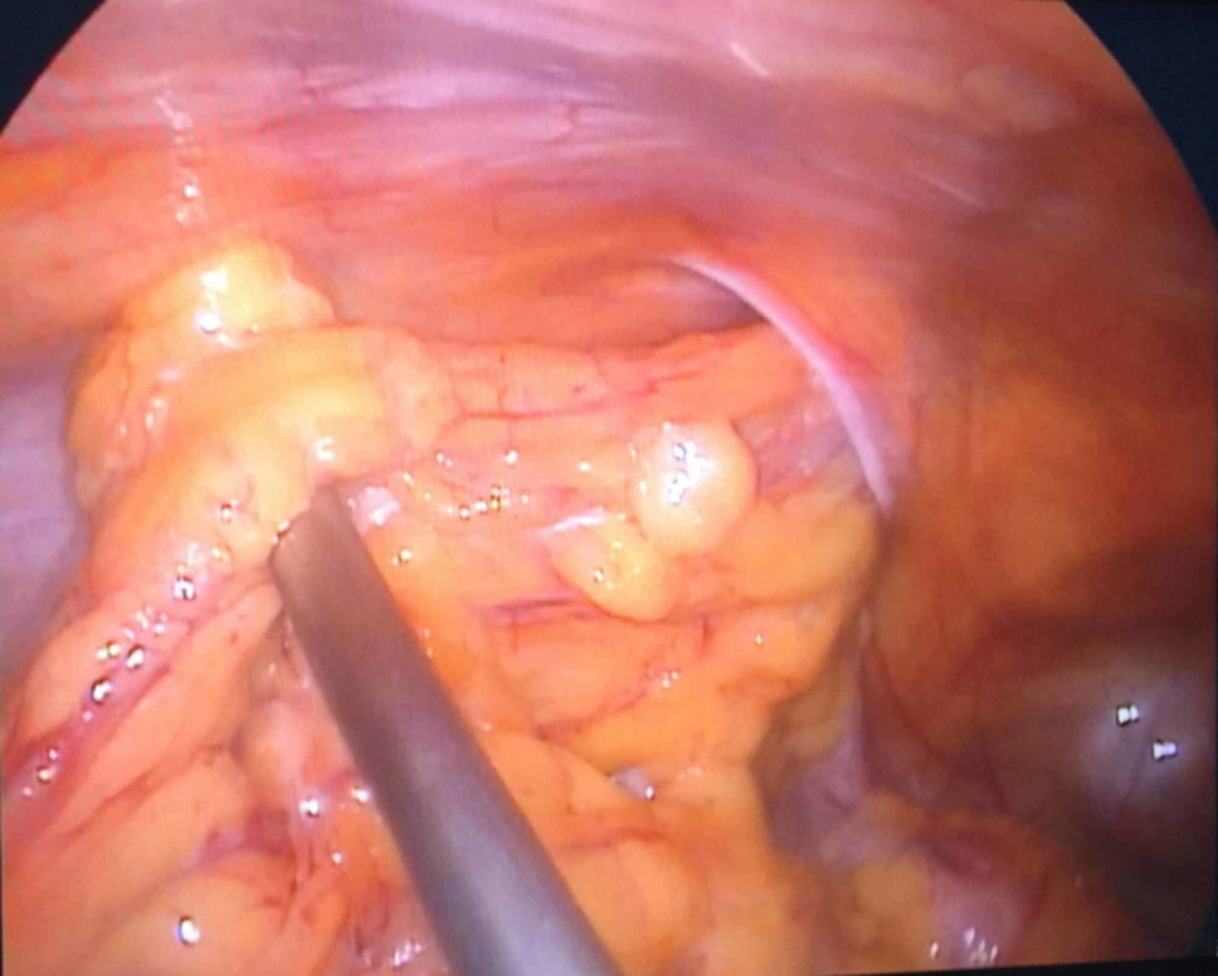
Incarcerated hernia

A total of 31 consecutive male patients with inguinal hernias were prospectively recruited to undergo TAPP repair. There were a total 34 hernias, including 11 indirect inguinal hernias, 19 direct hernias (Figure 5), 4 pantaloon hernias (combined direct/indirect inguinal hernia), and 4 recurrent hernias. The four recurrent hernias developed after open anterior hernia repair. The mean age was 60.4 ± 11.8, and 96.8% of cases were male. Strangulated hernias and incarcerated hernias accounted for 2.9% and 8.8% of cases, respectively (Figure 6). There were 17 cases of right-sided inguinal hernia (54.8%), 11 cases of left-sided inguinal hernia (35.5%), and 3 cases of bilateral inguinal hernia (accounting for 9.7%). The demographic and characteristics of the hernia data are shown in Table 1.

**Table 1:** Baseline characteristics of hernia patients

Variables	31 patients with 34 inguinal hernia
Age (mean, range)	60.4 ± 11.8 (47–88)
Sex	
Male	30/31 (96.8%)
Female	1/31 (3.2%)
ASA classification	
Grade I	23/31 (74.2%)
Grade II	8/31 (25.8%)
Bilateral hernia	3/31 (9.7%)
Recurrent hernia	4/31 (12.9%)
Hernia category	
Uncomplicated	30/34 (88.2%)
Incarcerated hernia	3/34 (8.8%)
Strangulated hernia	1/34 (2.9%)
Hernia type	
Direct hernia	19/34 (55.9%)
Indirect hernia	11/34 (32.3%)
Mixed (direct and indirect)	4/34 (11.8%)

The repair duration of unilateral inguinal repair and bilateral inguinal were 57.1 ± 17.3 minutes and 80.3 ± 10.6 minutes, respectively. All patients underwent endotracheal anesthesia. During surgery, all Prolene mesh placed were of adequate size. No patients were converted to traditional open anterior hernia repair. Furthermore, no respiratory, cardiac, or neurologic complications happened intraoperatively. The mean duration of the postoperative stay was 3.9 ± 1.4 days. One (3.2%) case with contralateral inguinal hernia was detected intraoperatively. The postoperative complications are shown in Table 2. An early and three-month postoperative evaluation showed that 93.5% and 96.8% cases were categorized as “very good”, respectively. At the three-month evaluation, one case was reported with sensation disorder of the inguinal area, and there was no recurrence (Table 3).

**Table 2:** Operative duration and postoperative complications

Variables	All patients (n = 31)
Procedure time (mean, range):	
Unilateral hernia	57.1 ± 17.3 minutes (20–110)
Bilateral hernia	80.3 ± 10.6 minutes (75–125)
One-day postoperative pain	
Mild pain	13 (41.9%)
Moderate pain	18 (58.1%)
Postoperative hospital stay (mean, range)	3.9 ± 1.4 days (2–7)
Postoperative complication	
Subcutaneous emphysema	2 (6.4%)
Urinary retention	1 (3.2%)
Funiculitis	1 (3.2%)

**Table 3:** Short-term follow-up

Postoperative follow-up	All patients (n = 31)
Time to return to normal preoperative activities	
0–7 days	7 (22.6%)
8–14 days	23 (74.2%)
15–21 days	1 (3.2%)
One-month outcome	
Groin pain	2 (6.4%)
Recurrence	0 (0%)
Very good (uncomplicated)	29 (93.5%)
Three-month outcome	
Groin pain	1 (3.2%)
Recurrence	0 (0%)
Very good (uncomplicated)	30 (96.8%)

## Discussion

We designed this study to assess the feasibility and safety of TAPP hernia repair using Prolene mesh. The use of Prolene mesh, based on the tension-free concept, was a major breakthrough in the repair of inguinal hernias. It is now used in most hernia repairs in adult patients since popularized by Lichtenstein and improved by other surgeons. Gilbert et al. [8] developed the surgical technique by using the mesh with a three-dimensional theoretical effect to strengthen and maintain the posterior wall of the inguinal canal without tension, covering the myopectineal orifice.

Our results showed a low incidence of postoperative complications. The TAPP technique for hernia repair using Prolene mesh is feasible, without any special difficulties, even for the repair of large hernias, complex hernias such as pantaloon hernias, and recurrent hernias.

The average age of the research group was 60.4 ± 11.85 years (range: 47-88 years). Our results are similar to those of Peitsch’s study, with an average age of 59.1 years [9], and Tolver’s study with an average age of 55 years (range: 20-85 years) [10]. Most authors studying inguinal hernias agree that the incidence of inguinal hernia increases with age. In addition, the elderly are susceptible to comorbidities that cause increased abdominal pressure such as chronic cough, benign prostatic hyperplasia, and chronic constipation, which can create favorable conditions for inguinal hernias to occur.

Four patients in our study had received inguinal hernia surgery prior (accounting for 12.9%), including one patient who had been treated with TEP on one side two years earlier. Two patients underwent Lichtensten surgery, and one was treated with Shouldice surgical technique.

Inguinal hernias occur more often on the right side than the left side [11]. We recorded 17 cases of right-sided inguinal hernia (54.8%), 11 cases of left-sided inguinal hernia (35.5%), and 3 cases of bilateral inguinal hernia (9.7%). According to Peacock and Madden, there is a change in weight, muscle, and cornea due to a decrease in the synthesis and increase in the degeneration process of collagen, which weakens the structure of the inguinal canal so that it is easy to cause an inguinal hernia [12].

In 31 patients with 34 cases of hernias, we recorded 30 (88.2%) cases of primary inguinal hernia and 4 (11.8%) cases of recurrence. Among them, two cases were recurrence after surgery by the Lichtenstein method, one was after Shouldice surgery, and one was detected a lateral inguinal hernia after surgery to treat an inguinal hernia with TEP. During the surgery, all four cases of recurrence had good surgical results, with no complications occurring. With these four recurrences, the process of creating the cavity, and placing the mesh, the anatomical structure remains the same, as in the other primary cases. In the viewpoint of Tantia et al. [13], the use of laparoscopic surgery to perform surgical treatment in patients with recurrent inguinal hernia (after open surgery) will have the following three main benefits: the first is reduced postoperative pain for the patient, the second is the artificial plate placed in the right peritoneal cavity, where the hernia sac appears first, and the third is that with surgery, coming in from the back and having to reopen the scarred area of the front incision will be avoided.

In our study, four patients with inguinal hernia had preoperative complications (11.8%). Among them, one patient with a strangulated hernia who was admitted earlier than six hours (3.2%) was assigned for emergency surgery, three patients with incarcerated inguinal hernia (8.6%) were assigned for surgery according to the next plan. Cases of inguinal hernia with complications were carefully evaluated for hernia, and 100% of hernias were returned to the peritoneum after combined manipulation; there were no cases of herniated necrosis. According to Leibl et al., TAPP surgery effectively treats both complicated and uncomplicated hernias [14].

The mean time of the surgical procedure for unilateral inguinal hernia is 57.1 ± 17.3 minutes (range: 20-110 minutes) and that for bilateral inguinal hernia is 80.3 ± 10.6 minutes (range: 75-125 minutes). Our results of the unilateral inguinal hernia surgery time are nearly the same as those in Yang and Liu’s study of TAPP, at 54.0 ± 18.8 minutes [5], whereas Ciftci et al. [15] and Leibl et al. [14] reported 55 minutes. The reason is that TAPP has a better view of the inguinal anatomy. Therefore, it is easy to operate and shortens the surgical procedure time.

During the surgery, we found two cases with a contralateral indirect inguinal hernia, which were not yet detected by clinical examination as well as subclinical investigation. There were no patients converted to traditional open anterior hernia repair. Furthermore, no complications of respiratory, cardiac, or neurologic occurred intraoperatively.

Early complications after surgery occurred in four patients. Of these, one (3.2%) patient had urinary retention, one (3.2%) case had funiculitis, and two (6.4%) cases had subcutaneous emphysema. We managed these complications with internal medicine and physical therapy and followed up by clinical examination as well as ultrasound findings. All of them were finally discharged without any troubles.

The results of our study showed that patients had a short postoperative time and that they could return to normal preoperative activities shortly. On assessment using VAS on day 1 after surgery, there were six cases of moderate pain, accounting for 19.4%, and 25 cases of mild pain, accounting for 80.6%.

The mean time of postoperative hospital stay was 3.9 ± 1.4 days (range: 2-7 days). Our study results have the equivalent of postoperative hospitalization compared with the study of Yang and Liu, 3.9 ± 1.1 days [5]. In our view, laparoscopic surgery is less severe, with less pain after surgery, and has a shorter recovery time for individual activities and thus patients can be discharged earlier. We confirmed that TAPP surgery is a safe method and reduces postoperative pain and the recovery time after surgery.

At the time of the one-month follow-up examinations, the time to return to normal activities after hospital discharge was 0 to 7 days in 7 (22.6%) patients and 8-14 days in 23 (74.2%) patients; one case took three weeks of recovery. Thus, the majority of patients return to normal activities at the time of the second week after discharge (8-14 days), which is similar to the result reported by Sharma et al., 11.8 ± 2.35 days [16]. At three months, all patients had no recurrent hernia, and there was a slight numbness in the scrotum area.

## Conclusions

Laparoscopic TAPP surgery for inguinal hernia repair using Prolene mesh has shown many advantages such as high aesthetics, short hospital stay, reduced pain after surgery, and fewer complications. TAPP can treat common inguinal hernias, complicated inguinal hernias, and recurrent inguinal hernias, and can resolve combined peritoneal diseases. TAPP should be encouraged to be widely deployed in Vietnam.
